# Biochemical studies of some diagnostic enzymes in myocardial infarction

**DOI:** 10.1186/cc9423

**Published:** 2011-03-11

**Authors:** M Samir, H Khaled Nagi, D Ragab, M Refaie

**Affiliations:** 1 Cairo University, Cairo, Egypt

## Introduction

Myocardial infarction (MI) is a key component of the burden of cardiovascular disease (CVD). The main causal and treatable risk factors for MI include hypertension, hypercholesterolemia or dyslipidemia, diabetes mellitus, and smoking. Acute MI results in cellular necrosis with release of constituent proteins into the circulation. Measurement of specific enzymes has become an important clinical tool for the diagnosis and management of MI. The aim of this study was to demonstrate the role of arginase and adenosine deaminase (ADA) in patients suffering from MI, and in a group of patients with chronic renal failure (CRF) with cardiovascular diseases (CVD).

## Methods

In this prospective study including 90 consecutive subjects were included the MI group (GI) consisting of 30 patients with mean age = 51.7 admitted to critical care medicine (CCM) in Cairo University Hospital, Egypt. (GII) included 30 patients of the CRF with CVD group with mean age = 49.1 undergoing periodic hemodialysis three times per week, compared with 30 normal volunteers included as the control group.

## Results

The mean value of serum arginase enzyme activity in the control group was 27.9 ± 4.59 U/l. In (GI) the mean value was 70.42 ± 11.9 U/l. On the other hand, the activity of serum arginase enzyme in patients with CRF with CVD has mean value 32.43 ± 6.5 U/l, *P *< 0.05 compared with the control group. ADA in the control group was 20.1 ± 2.39 U/l. But in (GI) the mean value was 44.99 ± 9.4 U/l, indicating a highly significantly increase was observed as compared with the control group (*P *< 0.001). The activity of ADA in CRF (GII) was also high (59.83 ± 9.8 U/l; *P *< 0.001).

## Conclusions

ADA may be considered good diagnostic enzymes in patients suffering from MI, and ADA for patients with CRF with CVD.

